# ‘Getting through’ not ‘going under’: A qualitative study of gender and spousal support after diagnosis with colorectal cancer^☆^

**DOI:** 10.1016/j.socscimed.2009.01.004

**Published:** 2009-03

**Authors:** Carol Emslie, Susan Browne, Una MacLeod, Linda Rozmovits, Elizabeth Mitchell, Sue Ziebland

**Affiliations:** a MRC Social & Public Health Sciences Unit, 4 Lilybank Gardens, Glasgow G12 8RZ, UK; b General Practice & Primary Care, Division of Community Based Sciences, Faculty of Medicine, 1 Horselethill Road, University of Glasgow, Glasgow G12 9LX, UK; c Qualitative Research, 16 Tennis Crescent, Toronto, Ontario M4K 1JE, Canada; d Department of Primary Health Care, University of Oxford, Old Road Campus, Oxford OX3 7LF, UK

**Keywords:** UK, Gender, Care, Cancer, Spousal support, Emotional labour

## Abstract

Many studies have found that people with cancer value family support. Feminist work suggests that women carry most responsibility for practical and emotional support in families, but few qualitative cancer studies explicitly incorporate a gender perspective. We undertook secondary analysis of in-depth interviews with 33 married or cohabiting respondents with colorectal cancer in the UK to compare men and women's accounts of ‘spousal’ support. Both men and women described the vital role that their partners played in providing emotional and practical support. Mutual support and reciprocity were also key features of narratives; both men and women reported controlling their emotions to protect spouses and preserve ‘normal’ household routines. Traditional gender roles had some influence; some women organised ‘cover’ for domestic work and childcare when they were ill, while some men focused on making sure that their families were financially secure and partners were ‘protected’ from the effects of their stomas. Our findings illustrate the complexity of gendered constructions and performances of ‘care’ and contribute to debates about gender and emotional labour.

## Introduction

The diagnosis, management and treatment of cancer occurs within the context of complex social networks. People with cancer consider family members – especially partners or spouses – to be particularly important; they provide valuable emotional and practical support, ask questions and recall important details at consultations, and help make critical decisions about treatment (Speice et al., 2000). In addition, good family communication (Edwards & Clarke, 2004) and high levels of perceived family support (Baider, Ever-Hadani, Goldzweig, Wygoda, & Peretz, 2003) are associated with lower psychological distress in cancer patients. Despite the long history of feminist work focusing on women's traditional role as carers, relatively little work on cancer has incorporated a gendered perspective on family support or ‘informal care’. We conceptualise gender as a dynamic set of socially constructed relationships (West & Zimmerman, 1987); thus, we explore similarities and differences between men and women and acknowledge diversity among men and among women. In this qualitative paper, we examine the relationship between respondents with colorectal cancer and their partners.

Early feminist work established that women typically take responsibility for family health: “caring demands both love and labour, both identity and activity…(and) tends to have particular consequences for the identity and activity of women” (Graham, 1983, p. 14). Recent work builds on this distinction between ‘labour’ and ‘love’, differentiating between ‘caring for’ (practical support such as help with dressing, washing, feeding, cooking, cleaning or transport) and ‘caring about’ (emotional support such as trying to understand other people's feelings, listening to problems, showing appreciation and attempting to maintain or improve others' psychological well-being) (Richardson, Ong, & Sim, 2007; Strazdins & Broom, 2004; Thomas, Morris, & Harman, 2002). James (1989) calls this latter form of care ‘emotional labour’, arguing that the management of emotions (one's own and other people's) is hard, skilled work. She suggests that women carry the main responsibility for emotional labour and that, most of the time, “the value of the labour is as hidden as the value of the routine management of emotion” (p. 28). A recent study of couples with young children (Strazdins & Broom, 2004) found that both men and women agreed that the female partner had a higher involvement in emotional work than the male partner. While most commentators agree that, on the whole, women perform more care than men, there is debate over whether styles of caregiving are gendered. For example, Carroll and Campbell (2008) question the notion that male caregiving is invariably instrumental while female caregiving is part of maintaining family relationships.

Few studies of people with cancer have tried explicitly to compare the experiences of women and men with cancer – or their partners – with regard to caregiving and support. This is partly because many focus on breast cancer or on sex-specific cancers such as prostate and testicular cancer. Some studies have found important gender differences. Two studies – a longitudinal quantitative study and a meta-analysis – found that women report more distress than men, regardless of whether they have cancer or are the partner of someone with cancer (Hagedoorn, Sanderman, Boks, Tuinstra, & Coyne, 2008; Northouse, Mood, Templin, & Mellon, 2000). Northouse et al. (2000) found that women (both colon cancer patients and spouses) reported more problems carrying out work, family and social roles and less marital satisfaction than men; female spouses, in particular, fared badly, and reported less social support from family and friends than patients or male spouses. The authors suggest that this could be because women are more comfortable than men about discussing emotional distress or because women are held responsible for juggling multiple roles and so experience more disruption and distress when illness occurs. In contrast, some qualitative work suggests that caring behaviours may not always follow gendered norms. For example, Hilton, Emslie, Hunt, Chapple, and Ziebland (in press) found that both men and women with cancer engaged in difficult emotional labour to protect the feelings of loved ones, while Thomas et al. (2002) reported that both men and women tried to stay ‘positive’ and ‘keep things normal’ for partners with cancer.

We attempt to fill this gap in the literature by comparing data from in-depth interviews with married or cohabiting men and women with colorectal cancer to explore their accounts of support (both from partner/spouse to cancer patient and vice versa). We chose to focus on colorectal (‘bowel’) cancer, as this is a non sex-specific cancer and a major cause of death among both men and women (Payne, 2007). Similar proportions of men and women are diagnosed at younger ages, but rates are higher among older men than older women (aged 50+) (Cancer Research UK Cancer Statistics, 2008). Five-year survival rates have doubled since the late 1970s, but still vary markedly by stage at diagnosis. Surgery is the main form of treatment and recovery involves a new focus on bowel habits and sometimes learning to live with a temporary or permanent stoma (where the bowel is diverted to empty through an artificial opening in the side of the abdomen into a watertight bag which is emptied manually) (Manderson, 2005). Qualitative work suggests that this illness profoundly affects people's identities as competent adults (Rozmovits & Ziebland, 2004) and leads to feelings of isolation (Taylor, 2001). Thus, colorectal cancer offers an opportunity to explore accounts of practical as well as emotional support among both men and women.

## Methods

We report a secondary analysis of narrative interviews originally collected for the DIPEx project (now renamed ‘healthtalkonline’ www.healthtalkonline.org) which gives widespread access to a wide range of personal experiences of health and illness (Herxheimer & Ziebland, 2004). The website is unique because the experiences presented are based on interviews collected and analysed by experienced qualitative researchers using rigorous methods approved by an UK Multi-centre Research Ethics Committee. While the website features short illustrative extracts of people's experiences, the full in-depth interviews from which these are taken are available for secondary analysis (e.g. Emslie, Ridge, Ziebland, & Hunt, 2006; Hilton, Hunt, Emslie, Salinas, & Ziebland, 2008; Seale, 2006).

In 2001 and 2002, LR interviewed 39 people – usually in their own homes – for the DIPEx colorectal cancer module. A maximum variation sample (Coyne, 1997) was recruited through general practitioners, hospital consultants and support groups and varied by gender, age, time since diagnosis, and Duke's stage of illness; thus, some respondents had been clear of cancer for many years, others were receiving treatment and several were receiving palliative care (see Rozmovits and Ziebland (2004) for further details). First, respondents were asked to tell their own story of developing cancer, with little interruption. The interviewer then used questions and prompts to ensure that particular issues were explored (e.g. the effect of illness on relationships, communication with doctors, sex and intimacy after diagnosis). All interviews were audio and/or video recorded with patients' written consent, fully transcribed and copyrighted to DIPEx for use in research, teaching and broadcasting. At this point, the original researchers (LR and SZ) analysed the data to identify emergent themes, wrote thematic topic summaries for the website, and developed papers (Rozmovits, Rose, & Ziebland, 2004; Rozmovits & Ziebland, 2004).

This paper aims to explore how men and women with colorectal cancer discussed the experience of support between themselves and their partner/spouse. For this secondary analysis, we, therefore, analysed the accounts of the subset of DIPEx participants who were married (*n* = 30) or cohabiting (*n* = 3) (for brevity, we sometimes use the terms ‘spouses’ or ‘spousal support’ in this paper). Our sample consists of 17 men and 16 women aged between 29 years and 76 years at diagnosis. Women were younger than men (mean age 49 years and 58 years, respectively, at diagnosis, and 55 years and 65 years at interview). Most respondents (15 men and 15 women) had children, but more women than men had dependent children (8 women and 4 men had children aged under 16 years at diagnosis). Not all the participants were asked directly about support from their spouse and how they had supported others, but all were asked questions that prompted replies describing experiences of support.

The transcripts were read repeatedly and CE recoded the raw data thematically, after discussion with SB, UM and EM about emerging themes in accordance with our focus on gender. All extracts of data relevant to spousal support were reviewed and summarised independently by three researchers. QSR Nvivo 2.0 was used to facilitate the analysis of themes and systematic comparisons across transcripts. Following the principle of the constant comparative method (Lincoln & Guba, 1985), each transcript was repeatedly compared across and within male and female groups to identify common themes. We present extracts from respondents' narratives to illustrate these themes (for each quotation, we give the respondent's ID number followed by ‘w’ to indicate the speaker is a woman or ‘m’ to indicate a man, and their age at interview). Particular attention was paid to deviant or contradictory cases. All authors discussed emerging themes, refined the analysis and commented on drafts of this paper.

## Findings

Rather than only conceptualising ‘care’ as something that partners provided to people with cancer, it was clear that mutual support and reciprocity were important features of narratives. For clarity, we have chosen to discuss support provided by partners first, and then describe support provided by respondents.

### Respondents' accounts of support from partners

Respondents were generally extremely positive about support from partners during their illness. Many suggested that they would have failed to have ‘got through’ or would have ‘gone under’ without their partner and a number felt that the experience strengthened their relationship. Partners acted as advocates with medical professionals, drove patients to appointments, researched information on the internet, canvassed medical friends and relatives for information and encouraged patients to regain fitness.

It was sometimes hard to separate physical (‘caring for’) and emotional (‘caring about’) support in accounts as they seemed inextricably linked. For example, CRC17w described how her husband demonstrated his love for her through emotional support and help with practical tasks. She was surprised by, and appreciative of, his level of care and felt that it aided her recovery. Similarly, CRC11m appreciated his wife's emotional support as well as her practicality:

*I realised then what…love is all about. He just accepted everything looked after me, did everything, did the cooking; he bolstered me up. He put up with the accidents I was having (with the colostomy), he helped me clean up, took my nighty off and did all those sort of things that really I would never have expected him to do. And…seeing that coming out in him helped me more…than any support from anybody else (CRC17w, 54 years).*

*My wife (supported me). She has the attitude of a nurse…that strong attitude. She was there all the time… We spoke about all these things openly. She was there when the stoma nurse was there, she was there…to look after me. Although I thought I could cope, she is the one (CRC11m, 60 years).*

Men and women emphasised different qualities when discussing emotional support. Men's descriptions of their wives suggested emotional strength, dependability and lack of fuss (‘brick’, ‘stalwart’, ‘strong attitude’, ‘doesn't fall to pieces’, ‘coped with it very well’). In contrast, women tended to focus on the upbeat nature of the support they received; husbands were repeatedly described as ‘optimistic’, ‘cheerful’ and ‘positive’. Both these forms of emotional labour (not making a fuss and remaining upbeat) could be argued to help to maintain normality and promote control (Richardson et al., 2007; Thomas et al., 2002).

Both men and women discussed how the illness challenged their identity as independent adults (a separate paper addressed this issue using data from all the respondents in the DIPEx module (Rozmovits & Ziebland, 2004)). Worries about overdependence on partners seemed more important in men's narratives than women's. Images of childish helplessness were contrasted (implicitly or explicitly) with adult competence and strength. The transition to child-like dependence was invoked by one man who described how his wife cared for him like a ‘nanny’. Other men described how upsetting it was when wives had to help them after accidents with colostomy bags. CRC11m explicitly referred to his identity as a man when he noted that he – not his wife – should be the ‘protector’:

*My wife is such a brick…she just…clears up, no tears…she's so strong and it made me upset to see her coping with me. I wanted to cope by myself and I couldn't. I think that's the hardest thing to watch someone you love looking after you… As the man you feel you're the protector, the provider… To see your wife washing your feet…taking your underwear off or cleaning you up. It's so humiliating (CRC11m, 60 years).*

*I remember…my wife…helping me to put pyjama bottoms on and this thing (bag) just leaked everywhere. All over the sheets, on the bed that had been changed and all over my pyjamas, and I just lay back and wept, wept like a child (CRC35m, 54 years).*

CRC26m also referred to his gender identity when he discussed how his wife offered too much practical support but not enough emotional support. His description of trying to be ‘a hero figure’ suggests (traditional masculine) traits of endurance, courage and self-reliance, and he also alludes to his mental strength and independence:

*She started off by wanting to do everything for me and I said ‘No, look, I've got to judge this for myself. I will ask you when I need help.’… But emotionally I was having to support her… I wish she'd been more open about it… In my arrogant way I set out to try to be a hero figure, you know, if I'm gonna die I wanna die gracefully but…try and fight this, I'm gonna try and stay alive… I believe I was 90% self-supporting. I've always been very independent and very strong mentally (CRC26m, 57 years).*

This account was unusual because a lack of emotional support in partners was more commonly discussed by women. Some women's narratives suggested they believed that some men *could* not – as opposed to *would* not – provide emotional support. Occasionally, this was because of a husband's own illness, but more often it was to do with the type of man they were perceived to be (e.g. ‘not really capable’ of giving emotional support). For example, CRC25w said:

*He's a very closed book really. He's one of these men that keeps himself to himself and doesn't show a lot of his emotions… I wish sometimes my son would talk to him more because I know men find it more difficult to open up and talk (CRC25w, 66 years).*

While most women emphasised how supportive their husbands had been, some also mentioned occasions when expressions of care had been problematic. For example, when husbands took a very optimistic approach, this could make it difficult for women to talk about their fears, particularly worries about death. In addition, CRC17w (also quoted above praising her husband's support) described how his desire to cook her ‘lovely meals’ after surgery was unhelpful, while CRC33w found her husband's need to exclude others difficult. These extracts illustrate the difficulty of dealing with others' needs when recovering from cancer:

*Although he was…showing me lots of love and support, he did find it quite difficult. He wanted to cook me lovely meals and I knew I couldn't cope with them… I didn't want to upset him, but at the same time I knew that I really HAD to think about myself and my own needs…and…they just had to deal with any problems that they had…on their own (CRC17w, 54 years).*

*He was very supportive…he…still continues to try to do everything for me. He was excellent but emotionally he didn't want anyone else near (me)…if anyone turned up (at the hospital) and he was there…it wasn't very pleasant because I knew he didn't really want them to be there, so that made it hard for me … And he would quite often end up being emotional when he left me, which wasn't very good for me either (CRC33w, 60 years).*

The problems some couples have in reconciling different coping styles have been discussed elsewhere. It is often argued that men prefer more pragmatic problem-focused coping strategies such as information-gathering, while women are thought to favour emotion-focused coping strategies such as mutual support (Gray, Fitch, Davis, & Phillips, 1996; Seale, 2006). The findings from some other studies challenge the simplicity of this assertion (Emslie et al., 2007; Hilton et al., in press; MacGeorge, Graves, Feng, Gillihan, & Burleson, 2004), but it is clear that for some (heterosexual) couples, gender differences in coping strategies cause distress which exacerbates problems during illness. The difficulties that partners' lack of communication or continual optimism caused some women in our study have been reported by others (Chesler & Parry, 2001). Reay, Bignold, Ball, and Cribb (1998) attribute these behaviours to the cultural expectation that men should comfort and protect their wives and be the ‘rock’ on which the family depends, leading men to suppress their own feelings and feel responsible for raising other people's spirits. As Northouse et al. (2000) suggest “…even though patients and their family caregivers share the same cancer experience, their ways and times of responding to it may differ, due in part to their different roles (i.e. patient vs. caregiver) and in part to their…responses as males and females” (p. 283).

Our findings should also be set in the context of near universal dualisms which equate women with nature and men with culture (Ortner, 1974). Women (as nature) are associated with a lower order of being and assigned “to the realm of the body, its fluxes and wastes” (Twigg, 2000; p. 407). Women have also traditionally been responsible for low status tasks in the household such as the removal of dirt, particularly cleaning dirty bodies and dealing with bodily waste, which has clear salience with regard to ‘caring for’ partners with colorectal cancer (Oakley, 1974). Thus, it is perhaps not surprising that women expressed surprise when men were willing to perform ‘gender inappropriate’ tasks such as intimate personal care.

### Respondents' accounts of the support they provided to partners

Reciprocity is an important component of supportive relationships, particularly between spouses. Thus, rather than being passive recipients of care from spouses, many narratives suggested that respondents regarded their relationships as mutually supportive ‘caring partnerships’ (Lynam, 1995; Richardson et al., 2007). For example, CRC2m described the reciprocity of emotional support between himself and his wife, while CRC11m, quoted above praising his wife's support, also discussed how he had supported her emotionally at a different stage in his illness (undergoing chemotherapy). Similarly, CRC23w described how different relatives' illnesses had taken priority at different times:

*My wife was distressed…because she's a more emotional person than I am and I think I probably try to be supportive to her. On the other hand, I guess there were times when I was distressed and she was supportive to me (CRC2m, 68 years).*

*My wife is such a brick, the things she's done for me… I had to keep saying ‘I'm strong, I'm fine’ and she was happy on the surface at seeing that. I had to very much steel myself, the emotional side, because…(when I was) going for the chemo, she was falling apart (CRC11m, 60 years).*

*(My husband) couldn't do without me and my son and my daughter-in-law needed my help (when my son was ill), but I need them as well (CRC23w, 68 years).*

Like their partners, respondents performed emotional labour to try to appear positive and strong. Both men and women described hiding their distress from their spouses by ‘putting on a brave face’ and trying to maintain ‘normality’. Parents, in particular, emphasised the importance of maintaining family routines. For example, CRC35m was determined to remain ‘part of this family’ rather than staying in bed, while CRC27w organised family birthday parties during her illness:

*It was my goal to get out of bed every day, no matter how difficult it was…make an effort to come down and still be part of this family. Just listen to what was going on, the children coming home from school and what concerns my wife had (CRC35m, 54 years).*

*I came home for the weekend (after being diagnosed) and had my son's birthday party the next day which was quite…a lot to handle but I was happy to handle it… It was my husband's 50th…ten days after I came out of hospital and by then I was already able to cope with putting together a party for a hundred people (CRC27w, 47 years).*

Respondents also recognised that their spouses felt helpless ‘on the sidelines’ and that the strength and optimism they projected took effort. Women described specific strategies they used to try to help their partners including: finding practical tasks so he could feel he was doing something positive to support her; making future plans to demonstrate to him that she believed she would survive; planning ahead further than she felt comfortable with because her husband needed a holiday; and asking to be moved from the hospital ward in which her mother-in-law had died as it upset her husband to visit her there.

Traditional gender roles influenced how respondents demonstrated ‘care’ for their families. Women with dependent children talked in some detail about the arrangements they had made for childcare and domestic labour when they were in hospital or recovering from surgery, but the few men with dependent children did not discuss this. For example, one woman described her extensive domestic preparations before going into hospital and how she resumed housework quickly after her return. Her account (“He's very good but…”) echoes other work about what can reasonably be expected of husbands with regard to domestic work (Connell, 2005):

*I was rushing round trying to do all the practical things…to leave things ready for when I wasn't there… He's (husband) very good but I had to sort the freezer out and…made a giant fruit cake because he likes cake, did all those little jobs and got my stuff ready and did a lot of washing and changed the beds. My husband is not the best, did his best, he had all the animals to look after and the children (CRC6w, 62 years).*

Some mothers recognised that it was important for their recovery to put personal needs before the needs of loved ones, but found this difficult. For example, one woman (CRC21w) discussed how she had moved from the ‘treadmill of servicing other people’ to prioritising caring for herself, while another (CRC39w) similarly acknowledged that her needs ‘got lost’ and resolved that she needed to treat herself better in future:

*It gave me permission to care for myself as the first priority…it was about the first time in my life that I can remember feeling…that looking after me is not selfish and…the children, the husband, the parents, they'll manage… I think I just got on (the) treadmill of servicing other people (CRC21w, 54 years).*

*I probably didn't spend enough time on me before. There was my son, there was my husband, there was my job and I may have got lost somewhere in the middle of all that and so I decided to be kinder to me in future (CRC39w, 51 years).*

Mothers with dependent children under 16 years stressed the importance of their caring role most strongly, but others made the same point. For example, one woman without children when diagnosed expressed concerns that her husband's needs were not being met because of her illness, while another woman with a sick husband and an adult son recovering from a stroke explained how she felt she *had* to get better as there were “too many people depending on me”. CRC25w had nursed her mother and her aunt, and her extract illustrates how perceiving oneself as a ‘carer’ could make it difficult for women to accept their own illness:

*I hate being in this position where the family are all troubled and bothered and not happy and it's all because of me… I've always been the one that's cared for everybody… It's not…‘me’ to be ill (CRC25w, 66 years).*

There was also some evidence that men's traditional role as providers influenced their narratives. Half the men talked about sorting out their finances so that their wives would have access to information while they were ill or their families would be provided for if they died, and their narratives suggested that they saw this as a form of care. Many men were, or had been, the main breadwinner and implied that they dealt with family finances as perhaps might be expected in this sample of older men. For example, CRC26m emphasised his competence in financial matters while CRC29m described how he wanted to make household paperwork accessible to his wife:

*I spent quite a bit of time sorting out my financial affairs to make sure that my wife and family would be comfortable…in the event of my death. And to write copious notes to my wife…who's got many great, great qualities but she's not the most numerate of people! (CRC26m, 57 years).*

*I had been throwing things like domestic bills and the gas servicing agreement into one awful pile. And so I felt that (it was) a priority… – and we did it together – to get them into a place where if for instance…the central heating failed while I was in hospital, she could just pull out the relevant piece of paper (CRCm29, 55 years).*

In contrast, few women talked about this subject. Those who did mentioned it in passing. For example, one woman (CRC25w) referred to her will as another thing she had ‘ticked off’ in response to a nurse warning her that things might not go well in hospital (“I've cleared out my knicker drawer and I've made my will and I've sorted out my funeral, there's nothing else I could do!”). Only one woman (CRC23w) – who, unusually, described herself as the ‘breadwinner’– raised concerns about her husband's ability to cope with finances:

*He's (husband) not worked for 26 years. He fell off a ladder…so I just…had to get on with my life and…be the breadwinner. That's the only thing I can say honestly say worries me, if anything happened to me…because I do everything. He hasn't a clue about the house, about money (CRC23w, 68 years).*

Finally, it appeared that the impact of a stoma on personal relationships differed for men and women. Around half the sample (7 women and 11 men) had had stomas. Both men and women described how repellent they had found stomas, at least initially, how the stoma had adversely affected their self image and how supportive spouses had been (Rozmovits & Ziebland, 2004). However, almost all of the women suggested that they had eventually come to terms with it to some extent and had resumed having sex. The very positive terms that women used to describe their partners (‘brilliant’, ‘wonderful’, ‘incredible’) suggested their surprise and pleasure when partners appeared to be less concerned about the stoma than they expected, and the importance of intimacy as a form of spousal support:

*I was very self conscious all the time…but my husband was absolutely incredible. As far as our intimate relationship goes he wasn't concerned… I was the one who was concerned… So it did add stress to our intimate relationship at the time…not from him but from me (CRC8w, 65 years, current stoma).*

*He doesn't bother about it… Sexually…if I'm getting undressed sometimes and he's there, I'll probably put my hand over my bag. That's what I used to do, I don't do it now…it probably took me longer to get to grips with (than) him (CRC20w, 37 years, current stoma).*

*He was very good about it…he didn't find it difficult to cuddle me. We did have intercourse during the time that I had the stoma and he saw it as something that was aiding me to get better as opposed to it being an invasion on my body he found unpleasant (CRC27w, 47 years, stoma in past).*

In contrast, men's narratives suggested that stomas had had a longer lasting and more negative effect on their personal relationships, but because respondents were not asked about their sex lives before their cancer diagnosis, we do not know if there were pre-existing gender differences. Men were also older than women in the study and it is possible that this played a part. Culturally dominant images of ‘masculinity’ (Connell, 1995; Courtenay, 2000) which emphasise strength and protecting others may also account for this gender difference. For example, one man (CRC11m) explicitly linked his difficulty in accepting his wife's offer to help with his stoma with his belief that ‘as the man, you feel you're the protector, the provider’ (quoted above). When describing how he had not had sex since his recent operation, he noted: “It's a very important part of everyone's life and particularly (as) a man you feel as though your manhood…has been sort of depreciated”. Other men suggested that their spouses needed to be protected from their stomas. For example, one older man who had had a stoma for 25 years (CRC4m), described how he was determined not to ‘bother’ or ‘upset’ his family, while CRC9m, who hoped his stoma would be reversed, had decided not even to discuss the subject of sex with his partner:

*I was quite determined that I wasn't going to upset the family with it (colostomy)… I don't think she (wife) could help me (with it) and I think it would appall her to try… I've kept it away all the time and dealt with it entirely myself (CRC4m, 87 years, current stoma).*

*There's no way that I've been near her since the operation because I feel, it's not dirty, but it's just in the way… She's said nothing but I didn't think it was fair to put her through a situation like that with me with a bag hanging down… I didn't want to give her an opportunity to refuse because I felt… I didn't want to put her in a position where she thought she was going to fail me in any way…maybe it would've made me feel worse. So better off not to bring it up, better off just to leave things as they were (CRC9m, 65 years, current stoma).*

Other powerful qualitative research confirms the difficulty of negotiating sex after stoma surgery. Kelly (1992) argues that, while in most social situations a stoma is not visible, its presence cannot be concealed during sex and this calls into question both private notions of self and public notions of identity. He suggests that managing the salience of the stoma in social interactions – so that it does not swamp ‘normal’ identities (e.g. adult, worker, spouse) – is a vital cognitive and practical task which counsellors and stoma therapists can assist with. Similarly, Manderson (2005) reflects on the disjunction between the idealized image of sex as *losing* control and the experience of sex for people with stomas who need to practice strict bodily control. Some respondents in her study suggested that their partners found it difficult to combine the roles of ‘lover’ and ‘carer’, and that assistance with intimate personal care sometimes coincided with partners' withdrawal from a sexual relationship. Thus, colorectal cancer brings to the fore taboos around bowel movements and sexuality and makes evident bodily processes which were not visible before the diagnosis: “existential emotions are evoked when the transparencies of bodily function, of mortality and of the strategies around which we have constructed our lives are suddenly made visible by the diagnosis of cancer” (Little, Jordens, Paul, Montgomery, & Philipson, 1998, p. 1491).

## Discussion

We found evidence of the influence of traditional gender roles in this sample. Some women described trying to organise ‘cover’ for domestic work and childcare when they were ill and returning to caring roles very soon after surgery. Others have found that traditional gender roles can interfere with women's recovery from cancer, as they try to juggle different responsibilities and find it difficult to prioritise their needs over the needs of their families (Emslie et al., 2007; Northouse et al., 2000). This is not straightforward, given the way that discourses around caring are gendered; for many women, caring responsibilities are closely intertwined with identity. Some men also linked accounts of their behaviour to traditional gender roles. Hegemonic forms of ‘masculinity’ emphasise strength, stoicism, the protection of ‘weaker’ women and children, control and success (Connell, 1995) and prescribe that “proper sexual activity must be initiated by a man” (Oliffe, 2005). Thus, men who emphasised their role as financial ‘providers’ for their families or those who described ‘protecting’ partners from stomas by choosing not to initiate sexual activity could be viewed as aligning themselves with hegemonic forms of masculinity.

However, to focus only on gender *difference* ignores similarities between men and women. First, both men and women described the vital importance of emotional and practical support from partners and recognised that this took energy and effort. Secondly, both men and women discussed putting considerable emotional labour into controlling their emotions to protect spouses, maintain household routines and preserve ‘normality’ for their families. Both personal accounts (Hutton, 2005) and research studies (Gray, Fitch, Phillips, Labrecque, & Fergus, 2000; Hilton et al., in press; Taylor, 2001) describe similar findings. Our study also adds to the evidence that caregiving between spouses – when either men or women are ill – should be perceived as a dynamic and reciprocal process (Richardson et al., 2007). Thirdly, similar qualities could be attributed to both men and women, undermining simple connections between gender and emotion. For example, men described themselves as strong protectors of their families, while also describing their wives as strong individuals who held the family together (Reay et al., 1998).

Our study has some limitations. First, most respondents were in their fifties or sixties at the time of interview and tended to occupy traditional gender roles. A study of younger respondents with more varied gender roles might have different findings. Secondly, we had data only from people with colorectal cancer. Further work with the partners of cancer patients would be helpful to identify whether their accounts correspond with our findings. Thirdly, these narratives were produced for a website with the intention of making personal experiences of colorectal cancer available to others – including family and friends. However, all of the respondents were given the option of anonymity on the website (and one third took this option), and given the opportunity to remove sections from their transcripts. This also raises the question of the extent to which qualitative data collected for one purpose can be used to answer a different question (Heaton, 2004). Our secondary analysis was appropriate because there was a good ‘fit’ between the research question we wished to address and the primary data; narratives focused in detail on the reactions of loved ones to the cancer diagnosis. However, it is important to acknowledge that areas such as sex and intimacy after cancer were discussed in less detail than they would be in a primary study of this topic. Finally, it is possible that, in the face of illness, the pressure to present accounts of a strong partnership and to depict themselves and their partners as fundamentally caring people could have led to less frank and more normative accounts. *What* men and women say about caregiving, and *how* they say it, can also be viewed as ways of performing gender (Carroll & Campbell, 2008). Thus, narratives which emphasise normative constructions of gender in which ‘women do the caring and men do the providing’ may be used to assert ‘moral worth’ (Blaxter, 1997; Reay et al., 1998).

Our findings contribute to debates about gender and emotional labour. First, while it is important to remember that the performance of emotional labour is set within the context of inequalities of power and resources (Duncombe & Marsden, 1998), the simplistic notion that male caregiving can be equated to practical or instrumental care while female caregiving is emotional or expressive can be challenged. Respondents did not consistently describe their partners' support in these gender stereotypical ways, and, indeed, often did not differentiate clearly between ‘caring for’ and ‘caring about’. Secondly, our work challenges the notion that women do – and men do not – perform emotional labour. Duncombe and Marsden (1998) neatly describe this simplification as ‘Stepford wives’ doing emotion work for ‘emotionally hollow’ men. Although women were more likely than men to report a lack of emotional support from partners, this was not the case for all women. It is also important to remember that suppressing or compartmentalising, as well as displaying and absorbing, emotions is a form of emotional labour (Craib, 1995; Reay et al., 1998; Williams, 2001). The former may be more commonly associated with hegemonic forms of ‘masculinity’ while the latter may be more commonly associated with culturally dominant forms of ‘femininity’. Thus, how we define emotional labour is important. Exley and Letherby (2001) warn against seeing emotion work as completely selfless, given that it also serves to reconstruct and reaffirm people's own identities. Thirdly, our work illustrates the complexity of gendered constructions, and performances, of ‘care’. For example, having sex when one partner had a stoma involved the negotiation of both practical ‘bodywork’ skills (Twigg, 2000) and emotional labour. Women's narratives suggested that they perceived that husbands demonstrated love and care through ignoring their stomas and continuing with sexual relationships, while men's narratives suggested that they demonstrated care and protected wives through refraining from initiating, or sometimes even discussing, sex.

In this study, both men and women described the important role that their partner played in their recovery from cancer. A recent systematic review of delay in colorectal cancer diagnosis (Mitchell, Macdonald, Campbell, Weller, & Macleod, 2008) suggests that social networks play a part in reducing patient delay (the time between first noticing a symptom and first consulting a doctor), as people seek advice from, or make help-seeking decisions based on the experience of, others. Thus, it is likely that spousal support plays an essential role not only following diagnosis, but also in the process of diagnosis itself, potentially facilitating earlier diagnosis and ultimately better disease outcomes. However, it cannot simply be assumed that appropriate support from partners will always be forthcoming.

Our findings may help to alert health professionals to the complexity of personal responses to a diagnosis of colorectal cancer. They may not realise the demands of ‘emotional labour’ as they support patients through diagnosis and treatment. Preparation for this aspect of a diagnosis, given by professionals such as specialist nurses, may help patients and their families. In this way, the ‘person’ rather than the ‘patient’ would be informed about what to expect from the disease. Secondly, our findings point to the importance of health professionals facilitating communication about sexuality and intimacy after cancer, in cases where they have established that the patient regards them as an appropriate person with whom to discuss these issues (Hordern & Street, 2007). Our findings also suggest the potential importance of couple counselling in this area. Finally, our work helps to remind health professionals that cancer diagnosis, management and treatment take place within the context of complex social networks and that the mutual support between patients and their partners is an important part of adjustment to the disease.
